# All-Trans Retinoic Acid Grafted Poly Beta-Amino Ester Nanoparticles: A Novel Anti-angiogenic Drug Delivery System

**DOI:** 10.34172/apb.2020.026

**Published:** 2020-02-18

**Authors:** Nadia Karimi, Kamaran Mansouri, Mohammad Soleiman-Beigi, Ali Fattahi

**Affiliations:** ^1^Department of Chemistry, Faculty of Basic Sciences, Ilam University, Ilam, Iran.; ^2^Medical Biology Research Center, Kermanshah University of Medical Sciences, Kermanshah, Iran.; ^3^Pharmaceutical Sciences Research Center, School of Pharmacy, Kermanshah University of Medical Sciences, Kermanshah, 6734667149, Iran.; ^4^Current affiliation: School for Engineering of Matter, Transport and Energy, Arizona State University, Tempe, AZ, USA.

**Keywords:** All-trans retinoic acid, Anti-angiogenesis, Nanoparticles, Poly (β-amino ester), Response surface methodology

## Abstract

***Purpose:*** Developing chemotherapy with nanoplatforms offers a promising strategy for effective cancer treatment. In the present study, we propose a novel all-trans retinoic acid (ATRA) grafted poly beta-amino ester (PBAE) copolymer for preparing nanoparticles (NPs).

***Methods:*** ATRA grafted PBAE (ATRA-g-PBAE) copolymer was synthesized by grafting ATRA to PBAE; it was characterized by proton nuclear magnetic resonance, Fourier transform infrared, and thermogravimetric analysis. ATRA-g-PBAE NPs were prepared by the solvent displacement method. Design-Expert software was employed to optimize size of NPs. The morphology was evaluated by transmission electron microscope, and ultraviolet-visible spectroscopy was applied for drug release. Cytotoxicity was evaluated toward HUVEC cell line, and the 3D collagencytodex model was used to evaluate anti-angiogenic property of PBAE, ATRA, and NPs.

***Results:*** The optimum size of the NPs was 139.4 ± 1.41 nm. After 21 days, 66.09% ± 1.39 and 42.14% ± 1.07 of ATRA were released from NPs at pH 5.8 and 7.4, respectively. Cell culture studies demonstrated antiangiogenic effects of ATRA-g-PBAE NPs. Anti-angiogenesis IC_50_ was 0.007 mg/mL for NPs (equal to 0.002 mg/mL of ATRA) and 0.005 mg/mL for free ATRA.

***Conclusion:*** This study proposes the ATRA-g-PBAE NPs with inherent anti-angiogenic effects as promising carrier for anticancer drugs with purpose of dual drug delivery.

## Introduction

Targeting a drug through selective polymeric nanoparticles (NPs) as a drug delivery system represents novel and systematic way for cancer therapy. This system has been widely used because of its ability, which includes biocompatibility, biodegradability, and prolonged circulation. Nanotherapeutic cancer therapy has been utilized to overcome several limitations of conventional drug delivery system such as poor oral bioavailability, lack of water solubility, high toxicity, low therapeutic indices, and inconsistency in circulation.^1^ Various drug carriers, e.g. liposomes,^2^ micelles,^3^ and polymeric NPs,^4^ have been applied for physical loading of anticancer drugs. On the other hand, a substantial amount of effort has been directed toward developing anticancer polymer-drug conjugates. Using drug-conjugated polymers for preparing cancer nanotherapeutics has been more appealing because of their inherent anticancer effects and potential ability for dual drug delivery. Not only does drug-polymer conjugation increase solubility, stability, bioavailability and efficacy of conjugated drugs,^5,6^ but it can also form NPs as a carrier for other drugs to modify pharmacokinetics of loaded drugs and to enhance their pharmacodynamics by synergistic effects; co-delivery of anticancer drugs can reduce drug resistance and enhance the efficacy of treatment.^7,8^

All-trans retinoic acid (ATRA) is a promising candidate for differentiation therapy of acute promyelocytic leukemia. It is also an effective drug for prostate and lung cancers. ATRA has critical roles in cell growth, differentiation or apoptosis of cancer cells, and immune function through binding to its nuclear receptors.^9^ Recently, the anti-angiogenic effect of ATRA has been evaluated. Angiogenesis is essential for tumor growth and metastasis, and it has been shown that without angiogenesis malignant epithelial tumors could not grow more than several cubic millimeters.^10^ Despite its valuable biological effects for cancer therapy, ATRA suffers from adverse effects, including neurotoxicity, dry skin, skin rash, headache, and poor water solubility, urging the need to overcome these side effects.^11^ In our previous studies, we evaluated ATRA-g-chitosan micelles to overcome these limitations. However, low solubility and unacceptable blood compatibility of chitosan together with high particles size and wide polydispersity of the nanomicelles were the disadvantages of this system.^12^ We had also evaluated the application of polyethylene glycol-polycaprolactone-polyethylene glycol nanomicelles for ATRA delivery, which has suffered from initial burst and fast release.^11^ To overcome these drawbacks and limitations, in the current study, we applied poly beta-amino ester (PBAE) to conjugate with ATRA and to make ATRA-g-PBAE NPs. PBAEs are biodegradable and biocompatible polymers synthesized by facile method of Michel reaction and usually in the solvent-free environment.^13^ PBAEs have low cytotoxicity and pH-responsive solubility profile,^14^ which make them an excellent candidate for gene delivery^15^ and cytoplasmic delivery of anti-cancer drugs in the form of nano-carrier.^16^

Particle size and surface charge of nano-carriers are key factors, which affect blood circulation time, tumor penetration, and cellular uptake of NPs.^17^ Due to small size of NPs, they can increase blood circulation times leading to greater opportunity to accumulate at tumor sites and increasing therapeutic efficacy.^18^ Therefore, optimizing the size of the nano-carriers for increasing drug delivery efficacy is challenging, and the effective analysis of the experimental data is a significant step in every experimental study.^19^ Response surface method (RSM) is one of the most important experimental design methods to overcome this challenge and could be a favorable approach in this study.^20^

In this study, ATRA-g-PBAE copolymer was synthesized, and its NPs were provided by the solvent displacement method. The size of NPs was optimized by RSM. The characterizations of ATRA-g-PBAE copolymer were performed, and anti-angiogenic effect of NPs was evaluated.

## Materials and Methods

### Materials

1, 6-Hexanediol diacrylate, 1, 4-aminobutanol (ABOL), ATRA, N, Nˊ-dicyclohexylcarbodiimde (DCC), Pluronic F-127, 3-(4, 5-dimethylthiazol-2-yl)-2, 5-diphenyl tetrazolium bromide (MTT) and 4-dimethyl aminopyridine (DMAP) were obtained from Sigma-Aldrich (St. Louis, MO, USA). Dichloromethane (DCM) was obtained from Merck (Merck KGaA, Darmstadt, Germany) dried by refluxing over calcium hydride at 60°C for 2 h and distilled immediately before use. Dried dichloromethane was stored over molecular sieves. Mineral materials as sodium azide (NaN_3_), potassium bromide (KBr), NaHCO_4_ and silica gel 60 (MESH 63-200) column and hydrochloric acid (HCl) were supplied by Merck. Tetrahydrofuran (THF), diethyl ether, chloroform (CHCl_3_), methanol (MeOH), ethyl acetate (EtOAc), acetone and toluene were supplied by Merck and used without further purification. Cytodex 3 microcarrier beads were purchased from Amersham Pharmacia Biotech (London, UK). The Cell culture medium, fetal bovine serum (FBS), trypsin and other cell culture supplements were supplied from Gibco (Gibco BRL, Paisley,Scotland, UK).

### Methods

#### Synthesis of PBAE

**Figure 1:**
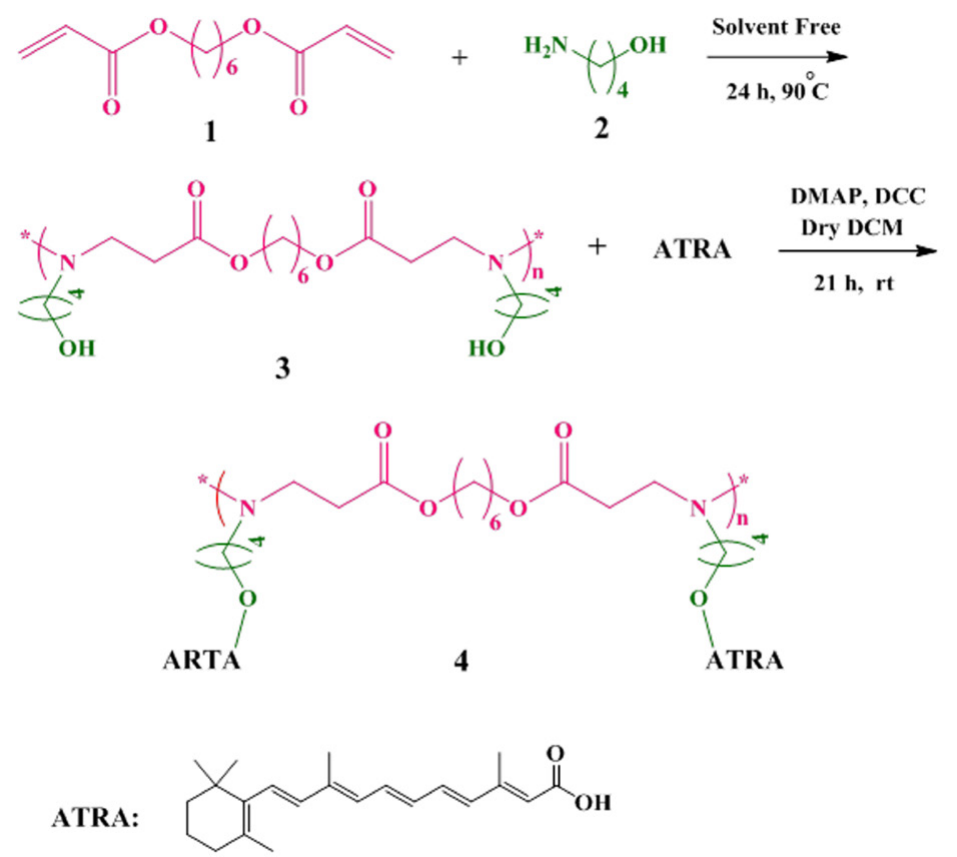
Synthesis of poly (β-amino ester) (PBAE) by reaction between 1, 6-Hexanediol diacrylate (1) and 1, 4-aminobutanol (ABOL, 2) under the solvent-free condition, and synthesis of all-trans retinoic acid grafted poly beta-amino ester copolymer (ATRA-g-PBAE copolymer, 4) by reaction between PBAE (3) and all-trans retinoic acid (ATRA) in presence of 4-Dimethyl amino pyridine (DMAP) and N, Nˊ-dicyclohexylcarbodiimde (DCC).

1, 6-Hexanediol diacrylate (271.44 mg, 1.2 mmol) (1) and ABOL (89.14 mg, 1 mmol) (2) were added to a dark reaction flask under solvent-free condition. The reaction mixture was stirred for 24 h and at 90°C under nitrogen atmosphere. Then, the reaction mixture was washed three times with 30 mL diethyl ether. The lower layer was separated and dried at room temperature (rt).^21^ The schematic of the reaction is shown in Figure 1. The structure of PBAE (3) was confirmed by FT-IR, TGA, and ^1^HNMR. The final PBAE was synthesized with a yield of 85%.

FT-IR (KBr): Ʋmax (cm^-1^) = 3421 (NH), 2600-3398 (OH), 2935 and 2862 (-C-H, aliphatic), 1732 (C=O, ester) 1577 (C-N), 1180 and 1053 (C-O, alpha ester and OH).

^1^HNMR (400 MHz; CDCl_3_): δ = 1.18, 1.53, 3.98 (6 H, CH_2_ of ring ester), 1.31, 1.57, 2.43, 3.49 (8 H, CH_2_ of ring ABOL), 1.89 (1 H, -NH- end ester), 2.38 (2 H, CH_2_ alpha –C=O), 2.73 (2 H, CH_2_ alpha –N), 3.83 (1 H, OH).

#### Synthesis of ATRA-g-PBAE copolymer

PBAE (315.41 mg, 1 mmol) (3), DMAP (105 mg, 0.787 mmol) and dry 15 mL DCM were added into a dark reaction flask. The reaction mixture was cooled and the temperature of the mixture was decreased to 0°C. ATRA (300 mg, 1 mmol) and DCC (206 mg, 1 mmol) were added, and the reaction mixture was stirred for 21 h under nitrogen atmosphere. Then, the solvent was evaporated with a vacuum rotary (Heidolph, Heizbad HB digit, Germany), and the precipitate was dissolved in 20 mL CHCl_3_. The reaction mixture was washed with 20 mL HCl (1 M) thrice and re-washed with 50 mL saturated sodium hydrogen carbonate. The residue was dried using anhydrous CaCl_2_ followed by filtration to remove the salt. The evaporation of the solvent was carried out in a rotary evaporator. The resulting product was purified by column chromatography through silica gel; in the first step, the CH_2_Cl_2_/EtOAc mixture (ratio of 1:1) and then the MeOH/CH_2_Cl_2_ mixture (ratio of 1:10) were used as mobile phases. The purified product was dried in a vacuum oven.^16^ The final ATRA-g-PBAE copolymer (4) was synthesized with a yield of 50%. The reaction is shown in Figure 1, and the structure was proved by FT-IR, ^1^HNMR, and TGA.

FT-IR (KBr): Ʋmax (cm-1) = 3325 (NH), 3024 (=C-H, alkene of ATRA), 2927 and 2862 (-C-H, aliphatic), 1728 (C=O, ester), 1647, 1577 and 1539 (C=C), 1361 (C-N), 1238 and 1153 (C-O, alpha ester).

^1^HNMR (400 MHz; CDCl_3_): δ = 0.742-1.01 (12 H, 6CH_2_ of ring PBAE), 1.04- 1.21 (8 H, CH_2_ of ring ABOL unit), 1.21- 1.69 (9 H, CH_3_ on cyclo ring ATRA), 1.98 (2 H, CH_2_ alpha –C=O of PBAE), 2.13-2.23 (8H, CH_2_ of cyclo ring ATRA), 2.27 and 2.74 (4H, 2 CH_2_ alpha –N), 3.98 and 4.14 (4 H, 2CH_2_O-of PBAE and ABOL unit), 3.63 (1 H, non-reacted OH of ABOL unit), 7.1-7.8 (8 H, 8CH of ATRA).

#### Characterization of ATRA-g-PBAE copolymer

##### FT-IR

The structure of PBAE, ATRA-g-PBAE, ATRA, and the interaction of different chemical groups were analyzed using FT-IR (IR prestige-21, Shimadzu Co., Japan) spectroscopy in the range of 4000-400 cm^-1^ at a resolution of 4 cm^-1^ and 25°C using the potassium bromide disk.

#### ^1^HNMR

The structure of the synthesized compounds was examined with the ^1^HNMR spectrum (Bruker, MSI, Karlsruhe, Germany). Chemical shifts are in part per million (ppm) downfield from tetramethylsilane as the internal standard, and samples were dissolved in CDCl_3_.

#### TGA

PBAE, ATRA, ATRA-g-PBAE were analyzed on the thermal analyzer (TA Instruments, STA 503, BAHR, Germany). For each analysis, about 5-10 mg of each sample was heated and the temperature was raised up to 700°C, at the ramp of 10°C/min with nitrogen as a purge gas.

#### Molecular weight

The molecular weight (MW) was determined using a Zetasizer instrument (Zetasizer, Nano-ZS, Malvern Instruments Ltd., Worcestershire, UK). Briefly, solutions of ATRA-g-PBAE copolymer were prepared at different concentrations; then average of dispersing intensity from six different concentrations of ATRA-g-PBAE solutions (5, 4, 3.33, 2.86, 2.50 and 1.25 mg/mL) was recorded using the molecular weight operating procedure. THF (the solvent of ATRA-g-PBAE copolymer) was used as the reference.

#### Characterization of ATRA-g-PBAE NPs

##### Preparation of ATRA-g-PBAE NPs

The NPs were prepared by the solvent displacement method. The solutions with concentrations of 2, 5 and 8 mg/mL of ATRA-g-PBAE copolymer were prepared in acetone. The organic phase containing ATRA-g-PBAE copolymer at the organic/aqueous phases volume ratios of 0.1, 0.14 and 0.18 was added dropwise to the aqueous phase, containing different concentrations of Pluronic F-127 under magnetic stirring at 25°C.^22^ Mean particle size and surface charge (zeta potential) of NPs were measured at 25 ºC using Zetasizer, and also effects of ATRA-g-PBAE copolymer concentration, volume ratios of organic/aqueous phases, and concentration of surfactant were optimized in order to achieve a desired average particle size for the experimental Design-Expert method.

#### Experimental design

The Design-Expert software (Version 8.0.7.1, stat Ease, Inc., USA) was used to optimize the size of the particles and achieve a set of designed experiments to investigate the effect of each parameter on the response function (size of NPs).^23^ Optimization of the size of NPs was performed using three selected factors affecting the particle size. The ATRA-g-PBAE copolymer concentration, organic/aqueous phase’s volume ratio, and surfactant concentration are those effective factors.

#### Measurement of surface charge and particle size

Particle size and surface charge (zeta potential) of NPs were measured at 25ºC using Zetasizer with a 632.8 nm He-Ne laser beam and 173° detection optics. The laser Doppler electrophoresis technique was performed to determine the zeta potential of NPs optimized by the design method using Henry equation. For this purpose, NPs were suspended in the deionized water and placed in an ultra-sonication bath for 30 s.

#### TEM

Morphology of NPs was observed with a Transmission Electron Microscope (TEM, Zeiss – EM10C – 80 KV, Germany). The NPs were placed on a carbon-coated copper grid and left at 25°C to dry.

#### Release study

The amount of the grafted ATRA to PBAE was determined by integrating the ^1^HNMR spectrum of the synthesized ATRA-g-PBAE copolymer using equation 1,^24,25^ and the amount of released ATRA was determined by a standard curve plotted using different concentrations of ATRA in phosphate-buffered saline (PBS, 0.001 N) containing 3% v/v ethanol at pH 5.8 and 7.4. NaN_3_ (0.1% W/V) was added to the release medium for preventing growth of bacteria and fungi. The amount of the dissolved ATRA was determined by UV-Vis spectrophotometer (UV-mini 1240, Shimadzu, Japan) at 365 nm.^26^

Eq. (1) $$\text{Grafted ATRA }\left(\text{\%}\right)\text{ = }\frac{\left(\frac{\text{Integration of CH from ATRA}}{\text{8}}\right)}{\left(\frac{\text{Integration of OCH2}\;\text{from PBAE}}{\text{8}}\right)}\text{×100}$$

To evaluate drug release, colloid solution of NPs (4 mL; containing 0.76 mg of PBAE and 0.43 mg of drug) was placed in a dialysis bag (molecular weight cut-off 3500). The dialysis bag was plunged in 100 mL of 3% ethanol in PBS at pH 5.8 or 7.4. The temperature was kept constant at 37°C with horizontal shaking at 100 rpm in a shaker incubator (NB-205, N-Biotek, Korea) for 21 days. In regular time intervals, 1 mL of the release medium was aspirated, and amount of drug release from each sample was assessed by the UV-Vis spectrophotometer. The aspirated medium was replenished with a fresh release medium.

#### Drug release kinetics

To ascertain the drug release mechanism of ATRA-g-PBAE NPs, the release data were ﬁtted using various kinetics models including ﬁrst order, Hixson-Crowell cube root, zero-order, Higuchi square root, and Kors-Peppas. Drug release kinetic from the ATRA-g-PBAE NPs was analyzed during the critical 21 days. The model with the highest squared correlation coefﬁcient (R_2_) and the lowest prediction error between the observed and the ﬁtted data was selected as the best model.^27^

#### Cell culture

HUVEC, a human umbilical vein endothelial cell line, was obtained from the Iranian biological resource center. Cells were cultured in a mixed medium of MCBD-131. Low passage (passages 3-5) HUVECs were used for this study.

#### Cytotoxicity assay

The cytotoxicity for 5 concentrations of ATRA (0.001, 0.002, 0.005 and 0.01 mg/mL), PBAE (0.002, 0.005, 0.01 and 0.02 mg/mL) and PBAE-ATRA NPs (0.003, 0.007, 0.015 and 0.03 mg/mL) was assayed on HUVEC cell line. The cell survival was studied using the MTT assay.^7^ 20 µL of ATRA, PBAE, and NPs in different concentrations were added to the wells. The plate was incubated for 48 h. After this period of time, the medium was removed and replaced by fresh medium containing 0.5 mg/mL of the MTT, and the plate was incubated for further 3 h. Then, the medium was removed and formazan crystals in each well were dissolved in DMSO (150 µL). Absorbance was measured using an ELISA plate reader (Synergy H1, Biotek, USA) at 540 nm and reference wavelength of 630 nm.

Percentage of viability was determined by dividing absorbance of each sample to control groups. The results were expressed according to the concentration required to kill 50% of cells as IC_50_.

#### HUVEC capillary tube formation in three-dimensional collagen gel

Cytodex 3 microcarrier beads were prepared according to the manufacturer’s instructions. HUVECs were mixed with the Cytodex 3 microcarrier beads by mildly shaking the cell suspension and micro-carriers every 20 minutes in MCDB-131 supplemented with 10% FCS for 4 h at 37°C and 5% CO_2_. Thereafter, the cell suspension with microcarriers was transferred to a 24-well tissue culture plate and incubated for 12-16 h under the same conditions. On the following day, the cell-incorporated beads were mixed with a collagen solution (collagen type I, 10 X MCDB-131, 23 mg/mL NaHCO_3_ and FBS with a ratio of 7.5:1:1:0.5, respectively) on ice, and 50 µL of the collagen solution was added to each well of the 96-well plate and allowed to rigidify in the incubator for 20 minutes at 37°C and 5% CO_2_.^28^ Afterwards, 250 µL of MCDB-131 with different concentrations of ATRA (0.001, 0.002, 0.005 and 0.01 mg/mL) and ATRA-g-PBAE NPs (0.003, 0.007, 0.015 and 0.03 mg/mL) were added. Effects of samples on sprout formation were investigated using Adobe Photoshop software (version 6.0), which presented the mean number of the sprouts in 20 beads for each treatment. We reported the percentage decrease in sprout formation three days after treatment compared to the control.

#### Statistical analysis

Three replicates were intended as means ± standard error of the mean (SEM) for biological assays and the data was evaluated by one-way analysis of variance (ANOVA) where appropriate. All statistical analyses were performed by Tukey’s test using Minitab software (version 18.1, SQC Institute, USA). *P* values <0.05 and <0.01 were considered statistically significant.

## Results and Discussion

### FT-IR spectroscopy analysis

**Figure 2:**
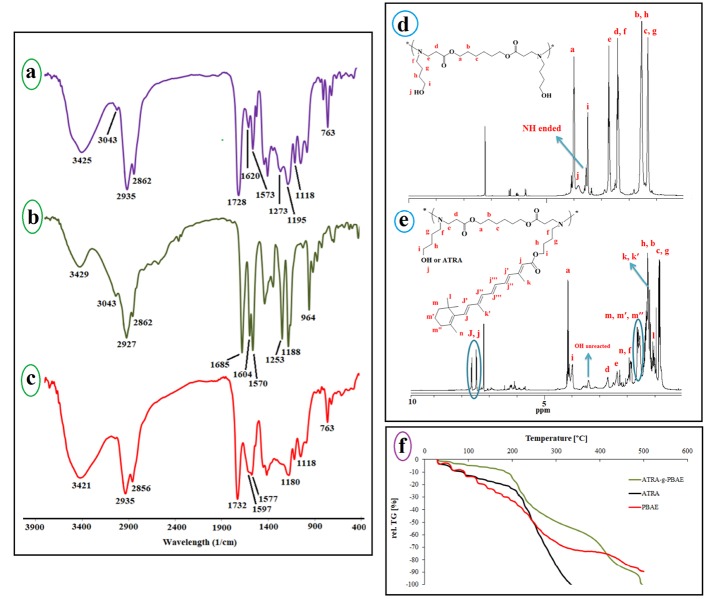
The FT-IR spectrum of ATRA-g-PBAE copolymer (a), ATRA (b) and PBAE (c), the ^1^H-NMR spectrum of ATRA-g-PBAE copolymer (d) and PBAE (e), and TGA curves for ATRA-g-PBAE, ATRA and PBAE copolymer (f).

The PBAE was synthesized from 1, 6-hexanediol diacrylate and ABOL by the Michel reaction between amine group of ABOL unit and double bond of 1, 6-hexanediol diacrylate. The FT-IR spectrum of the prepared PBAE (Figure 2a) indicated sharp bands at 3421, 2935, 2858, 1732, 1577, 1180 and 1053 cm^−1^ and the broadband at 3600-2500 cm^−1^. The peaks at 3421 and 1577 cm^−1^ corresponded to N-H stretching vibration and bending vibration of second amine of ABOL at the end of polymer chains, respectively. The broad peak at about 3600-2500 cm^−1^ was a feature of OH stretching vibration, and the peaks at 2858 and 2935 cm^−1^ corresponded to CH_2_ and CH_3_ symmetric and asymmetric stretching vibration aliphatic. The peak at 1728 cm^−1^ was a feature of C=O stretching vibration, and the peaks at 1180 and 1053 cm^−1^ were related to C-O stretching vibration of the ester. The FT-IR spectrum of the prepared PBAE indicated that the peak at 3105 cm^−1^, characteristic of C-H vinyl stretching vibration, and the peak at 1635 cm^−1^ corresponding to C=C vinyl stretching vibration were omitted in the PBAE spectrum, and the new band at 1577 cm^1^ appeared due to the reaction between amine group of ABOL unit and double bond of 1, 6-hexanediol diacrylate, converting the primary amine to tertiary and secondary amines. On the other hand, presence of a broad peak at 3600-2500 cm^-1^, related to OH, confirmed the reaction between 1, 6-hexanediol diacrylate and ABOL.

The ATRA spectrum (Figure 2b) showed peaks at 3429, 3043, 2927, 1685, 1604, and 1188 cm^−1^. The broad peak at 3600-2500 cm^−1^ was a feature of OH stretching vibration. The peaks at 3043 and 2927 cm^−1^ corresponded to C-H alkenes and aliphatic stretching vibration, and the intense band at 1604 cm^−1^ was related to C=C alkenes stretching vibration. The peak at 1685 cm^−1^ was a feature of C=O stretching vibration group of the carboxylic acid of retinoic acid, and the peak at 1188 cm^−1^ was related to C-O stretching vibration.

The FT-IR spectrum of the prepared ATRA-g-PBAE copolymer (Figure 2a) indicated sharp bands at 3325, 3035, 2927, 2862, 1728, 1647, 1539, 1238 and 1153 cm^−1^. The sharp peaks at 3325 and 1539 cm^−1^ were a feature of N-H stretching vibration and bending vibration of ABOL at the end of polymer chains. The sharp peak at 3035 cm^−1^ described C-H alkenes of retinoic acid and the peaks at 2927 and 2862 cm^−1^ corresponded to C-H symmetric and asymmetric stretching vibrations. The peak at 1728 cm^−1^ was a feature of C=O stretching vibration, and the peaks at 1153 and 1238 cm^−1^ were a feature of C-O stretching vibration of the ester. The presence of a peak at 1728 cm^−1^ correlated to C=O ester and the omitted peak at 1685 cm^−1^ corresponding to the carboxylic acid of ATRA can confirm the synthesis of ATRA-g-PBAE copolymer.

### ^1^HNMR spectroscopy study

^1^HNMR spectrum of PBAE (Figure 2d) indicates the sharp peaks at 1.3, 1.53, and 3.98 ppm, which can be ascribed to the methylene protons (-CH_2_- CH_2_-CH_2_-O-C=O) of the polymer backbone, and the peaks at 2.43, 1.31, 1.57, and 3.49 ppm can be ascribed to the methylene protons of ABOL side unit (-CH_2_- CH_2_-CH_2_- CH_2_-O-), respectively. There is also a peak at 2.38 ppm corresponding to the methylene protons vicinal to a carbonyl group (CH_2_C=O) and a peak at 2.73 ppm corresponding to methylene vicinal to an amine group (CH_2_-N). The peak at 3.83 is associated with the OH group of the polymer. In the ^1^HNMR spectra of PBAE, the peaks ascribing to the protons from alkenes of 1, 6-hexanediol diacrylate group (5-7 ppm) and the peak of protons from amine group (NH_2_, 2-3 ppm) of ABOL are omitted. Also, the presence of proton peak of hydroxide from ABOL at 3.38 ppm indicates the successful synthesis of PBAE.

^1 ^HNMR spectrum of ATRA-g-PBAE copolymer (Figure 2e) indicates sharp peaks at 7.1-7.8 ppm related to =C-H of ATRA, and the peaks at 4.14 and 3.98 respectively are attributed to protons of –O-CH_2_ chain of PBAE and ABOL unit. The peaks at 2.74 and 2.27 ppm correspond to protons of –CH_2_N chain of PBAE and ABOL, and the peak at 1.98 ppm is related to -CH_2_- protons vicinal to a carbonyl group. The peaks at 1.21-1.69 ppm can be attributed to the methylene protons of cycle ring and methyls of ATRA. The peaks at 1.04-1.21 and 0.742-1.01 ppm can be ascribed to the methylene protons of CH_2_ of ABOL and the backbone of the polymer, respectively. A shift in the =C-H peak of ATRA from 6.20 to 7.70 ppm due to resonance together with the omission of the peak at around 10 ppm, related to the proton of carboxylic acid group of ATRA, confirmed the synthesis of ATRA-g-PBAE copolymer.

### TGA study

Thermogravimetric analysis (TGA) is usually used to demonstrate the thermal stability of the polymers. TGA of dry samples of ATRA-g-PBAE copolymer, ATRA and PBAE at different heating rates were measured and the results were compared (Figure 2f). In the thermal degradation curves of all samples, the initial weight loss observed at temperatures below 100 °C was related to the evaporation of the absorbed water in the structure of samples. The second step with sharp weight loss is more important for explaining the thermal behavior of samples.

In the thermogram of PBAE, the second step started from 104 °C and continued to 195 °C and the degradation of polymer occurred with 17% weight loss in comparison to the initial weight of the sample. The PBAE molecule contains ester bonds (from 1, 6-hexanediol diacrylate monomer) and amine bonds; the ester bond is weaker and has less energy than that of the amine bond and breaks faster.

Degradation of ATRA started from 190°C and continued to 330°C and degradation of ATRA occurred with 89% weight loss compared to the initial weight of the sample; TGA curve of ATRA showed small weight loss (about 20%) at temperature of 190°C. The ATRA molecule contains a cyclohexenyl ring, a polyene chain containing conjugated double alkene bond, and a terminal carboxyl group. The weakened structure of the polyene chain (containing conjugated double alkene bond) in ATRA caused ATRA degradation at a low temperature. According to the ATRA-g-PBAE thermogram, behavior of ATRA-g-PBAE copolymer was similar to that of ATRA due to the presence of about 18% of ATRA in the structure of copolymer. The initial step started from 175°C and continued to 325°C and degradation of ATRA-g-PBAE copolymer occurred with 44% weight loss compared to the initial weight of the sample, where the degradation of the copolymer side chain (containing ABOL and ATRA) occurred. TGA curve of ATRA-g-PBAE copolymer compared to ATRA shows that degradation of ATRA-g-PBAE copolymer begins with a rate lower than ATRA, which can be related to conjugation of ATRA to PBAE. Third step of ATRA-g-PBAE copolymer degradation started from 360°C and continued to 480C; at this temperature, about 31% weight loss was indicated, where the degradation of the backbone copolymer occurred. At this level, the behavior of ATRA-g-PBAE copolymer was similar to that of the second step of PBAE.

### Molecular weight of polymer

Average molecular weight of ATRA-g-PBAE copolymer was calculated according to equations 2 and 3 given in the Malvern manual, and MW was 92.5 ± 6.99 kDa.

Eq. (2) $$K=\frac{2\pi \text{2}}{\lambda \text{4O }N\text{A}}\left(n_{\text{O}}\frac{dn}{dc}\right)\text{2}$$

Where, N_A_ is Avogadro, constant, R_g_ is Radius of gyration, λ_o_ is Laser wavelength, and n_o_ is Solvent refractive index.

Eq. (3) $$\frac{KC}{R\text{θ}}=\left(\frac{1}{M}+2A\text{2C}\right)P\left(\theta \right)$$

Where, *R*_θ_ is The Rayleigh ratio – the ratio of the scattered light to incident light of the sample, *M* is Sample molecular weight, A_2_ is 2^nd^ Virial Coefficient, *C* is Concentration, *P*_θ_ is Angular dependence of the sample scattering intensity. K is Optical constant.

### Experimental design method

Using the CCD design tool, RSM was applied for optimizing the size of the NPs and also analyzing the effect of each variable on the size of the NPs. Table 1 shows the results of 20 designed experiments suggested by the RSM method. According to the results, particle size of NPs was found in range of 138-247 nm. The response function was fitted by a quadratic polynomial model. The derived formula is demonstrated in terms of both actual and coded forms in equations 4 and 5.

**Table 1:** Factors in actual form

A* (mg/mL)	B**	C*** (mg/mL)	**Size (nm)**
5.00	0.14	0.60	163.3
5.00	0.10	0.60	149.4
5.00	0.14	0.60	162.2
8.00	0.10	0.00	217.5
8.00	0.14	0.60	189.1
8.00	0.10	1.20	156.9
8.00	0.18	1.20	176.2
5.00	0.14	1.20	160.7
5.00	0.14	0.60	162.7
5.00	0.14	0.00	185.1
8.00	0.18	0.00	247.5
5.00	0.14	0.60	175.1
5.00	0.18	0.60	168
5.00	0.14	0.60	160.5
2.00	0.10	0.00	147.5
2.00	0.18	0.00	158.8
5.00	0.14	0.60	164.9
2.00	0.10	1.20	138.6
2.00	0.14	0.60	147.8
2.00	0.18	1.20	145.5

* The concentration of ATRA-g-PBAE copolymer. ** The volume ratio of organic/aqueous phase. *** The concentration of surfactant.

Final equation in terms of coded factors:

*Size = +164.30 + 24.90 A + 8.61 B - 17.85 C - 13.71 AC + 9.13 C* ^2^ Eq. (4)

Final equation in terms of actual factors:

*Size = 96.79083 + 12.87083 A + 215.25000 B - 22.09306 C - 7.61806 BC + 25.36111 C* ^2 ^Eq. (5)

Where A, B, and C are the ATRA-g-PBAE copolymer concentration, organic/aqueous phases volume ratio, and surfactant concentration, respectively. According to the equations, all the experimental parameters significantly affect size of NPs. The *P* value of the model for all experimental parameters and interactions between variables is presented in the ANOVA table (Table 2).

**Table 2:** Analysis of Variance

**Source**	**Squares**	***df***	**Mean square**	**F value**	***P*** **value**	**Prob> F**
Model	12048.69	5.00	2409.74	65.15	<0.0001	Significant
A	6200.10	1.00	6200.10	167.64	<0.0001	
B	741.32	1.00	741.32	20.04	0.0005	
C	3186.23	1.00	3186.23	86.15	<0.0001	
AC*	1504.26	1.00	1504.26	40.67	<0.0001	
C^2^*	416.78	1.00	416.78	11.27	0.0047	
Residual	517.79	14.00	36.99			
Lack of Fit	379.79	9.00	42.20	1.53	0.3334	Not significant
Pure Error	138.01	5.00	27.60			
Cor Total	12566.49	19.00				

* The concentration of ATRA-g-PBAE copolymer and surfactant. ** The concentration of surfactant and surfactant.

**Figure 3:**
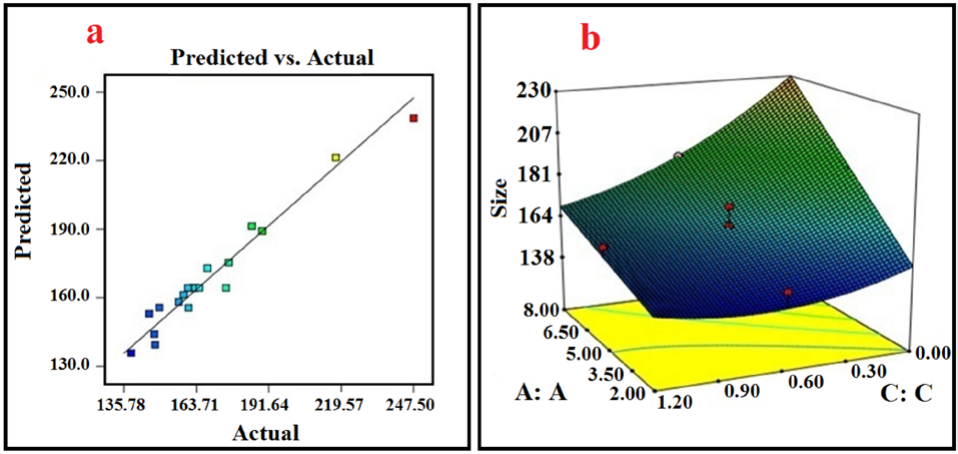
(a) Actual versus predicted values of Z-average by response surface methodology (RSM) model; (b) 3D plot of interaction between the concentration of ATRA-g-PBAE copolymer (A) and the concentration of surfactant (C).

The ANOVA table reveals the statistical significance of the model (all the statistical details which are necessary for analysis). The ANOVA table was used to analyze the variance (ANOVA) by calculating F-value. The lack of fit value of the model is 1.53 and is not significant, which confirms the accuracy of model. The coefficient of determination (R^2^), adjusted R^2^, predicted R^2^ of the model are 0.96, 0.94, and 0.90, respectively. These values are extremely adequate and show the precision and reliability of the model. Another way for ascertaining the precision and reliability of the model is to evaluate the prediction versus actual distribution plots for obtained data. As illustrated in Figure 3a, the line is very close to the 45-degree one and indicates that the regression suggested for data extrapolation has highly precise prediction capability.

According to the ANOVA table, all the three parameters and the interaction between parameters A and C indicate a significant effect on the size of NPs with *P* value <0.05. Figure 3b shows the response surface 3D diagram exhibiting effect of interaction between A and C and variation in size of NPs with these parameters. In the 3D response surface diagram, interaction of two variables is obtained simultaneously, while the third one is in its middle-level value.

### Effect of processing variables on the NPs size

The NPs were prepared by displacement methods. According to the data obtained from the ANOVA and equation 4, all the three independent variables contributed significantly to the size of NPs (*P* < 0.05). Furthermore, the larger coefficient of parameter A (concentration of ATRA-g-PBAE copolymer) in equation 4, in comparison to other coefficients, revealed that ATRA-g-PBAE copolymer concentration (A) among these three parameters had the most significant effect on the size of NPs. As illustrated by Table 1, the best result (smaller size) could be obtained when lower ATRA-g-PBAE copolymer concentration (2 mg/mL) and average levels of surfactant concentration (1.20 mg/mL) were chosen. According to the data obtained experimentally (Table 2) and the sign of the coefficients in equation 4, the trend in variation of the NPs size with variables could be justified. An increase in the ATRA-g-PBAE copolymer concentration resulted in the enhancement of the viscosity and gave rise to the generation of larger NPs. In the solvent displacement method employed in this study, the governing mass transfer phenomenon led to diffusion between aqueous and organic solutions. The rate of the diffusion directly depended on the viscosity of the organic solution, and decreasing the rate of the diffusion led to the generation of larger NPs. An increase in viscosity was followed by a decrease in the diffusion of the solvent to the outer aqueous solution and consequently the production of the larger particles.^29^

Another key parameter influencing the size of the NPs in this study is the organic/aqueous solutions volume ratio. Increasing the organic/aqueous solution volume ratio increases the NPs size. Furthermore, increasing this ratio reduces the mass transfer driving force and decreases the rate of diffusion mass transfer. On the other hand, as corroborated by other studies, decreasing this ratio can improve diffusion of organic solvent and increases the distribution efficiency of the organic phase into the external phase, leading to formation of smaller NPs. Additionally, the solvent diffusion into water allows the ATRA-g-PBAE copolymer particles to make contact with the water in which they precipitate, creating a nucleation point and causing NPs to grow.^30^

Increasing the surfactant concentration could have an inconsistent effect on the size of the NPs. At higher surfactant concentrations, agglomeration of NPs is prevented, but the viscosity of the aqueous solution is also elevated, which increases size of NPs. Based on such observations, high surfactant concentrations must be avoided. In this study, in surfactant concentrations up to 0.8 mg/mL, viscosity of aqueous phase had a certain effect on size of NPs, and increasing viscosity of aqueous phase caused an increase in the size of the NPs. Optimization of surfactant concentration is also important for improving biocompatibility of NPs and reducing production cost as in high concentrations, the surfactant residues in the colloidal solution can increase cytotoxicity, and removing it from the formulation is costly.

### Optimization and validation of the model

The size of the NPs was optimized using the RSM method. The optimization criteria were based on minimum size of NPs and surfactant concentration in range of 0.6-1.2 mg/mL. According to the mentioned criteria, some solutions and optimized conditions could be obtained by the software. Based on these results, the optimum conditions were determined; optimum size of NPs was found to be 137.93 nm for 2.97 mg/mL of ATRA-g-PBAE copolymer and 0.71 mg/mL of surfactant at organic/aqueous phase’s volume ratio of 0.1. The selected conditions were used in the validation study, with three experiments being carried out under such conditions. The average size of the NPs was 139.4 ± 1.41 (nm) under the optimized conditions. The prediction error was found at 0.01 (<0.05), which confirms the validity of the model. Measured zeta potential of optimized formulation was +5.12 ± 10.3 mV. It should be noted that PBAE with amine functional groups shows a positive surface charge, owing to protonation of amine groups. However, we observed a low positive charge (for zeta potential), as compared to other studies due to the difference in pH, the used buffer, and the percentage of the unreacted OH- ions of ABOL unit on surface of NPs.^31^

### Morphology of NPs

**Figure 4:**
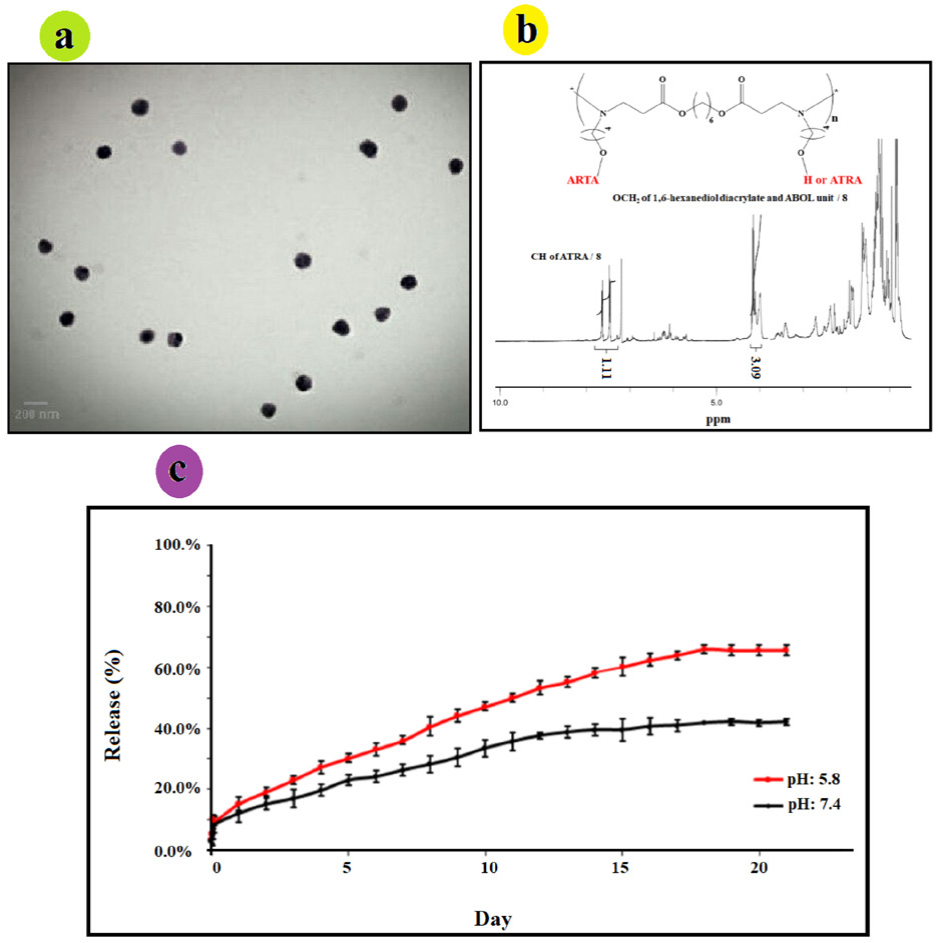
(a) TEM micrograph of ATRA-g-PBAE NPs, (b) ^1^H-NMR spectrum of ATRA-g-PBAE copolymer; the peak integrals related to CH_2_ group of PBAE and the peak integral of CH groups of ATRA, and (c) ATRA release curve from ATRA-g-PBAE NPs at 37°C.

The morphology of the NPs was determined by transmission electron microscope (TEM) at 2000 x magnification. Optimum condition of NPs was chosen: ATRA-g-PBAE copolymer concentration of 2.97 mg/mL, organic/aqueous phases volume ratio of 0.1, and surfactant concentration of 0.71 mg/mL. TEM micrographs of NPs indicate a spherical shape without any aggregation and adhesion. The TEM image is shown in Figure 4a. Size of NPs measured by ImageJ software was found to be 141.2 ± 4.7 nm, with the measured average size confirming their narrow size distribution. Results of TEM and DLS are in good agreement.

### Degree of substitution

Substitution degree of ATRA was evaluated by ^1^HNMR spectrum (Figure 4b) of ATRA-g-PBAE copolymer. The amount of grafted ATRA in ATRA-g-PBAE was determined using the peak integrals related to CH_2_ groups of PBAE to the peak integral of the CH groups of the retinoic acid in the ^1^HNMR spectrum of ATRA-g-PBAE copolymer. Calculated amount of graft was performed to determine release percentage, and the results demonstrated that grafted percentage of ATRA to PBAE copolymer was 36%.

### Release study

Release of ATRA from NPs was studied using dialysis method in PBS (0.001 N) at pH 5.8 and 7.4 as release medium. Results are illustrated in Figure 4c, indicating that the drug was released slowly at pH 7.4 (physiological pH), and only 42.14% ± 1.07 of the grafted ATRA was released after 21 days. Release of ATRA became faster at pH 5.8 (tumors and endosome pH), and 66.09% ± 1.39 of the grafted ATRA was released after 21 days.

The release curves of the samples demonstrated that release of incorporated ATRA from NPs at pH 5.8 and 7.4 was about 15.3 and 12.15%, respectively in 24 h. In the first 7 days, it was about 36.1 and 26.39%, and up to 58.2 and 39.51% were released after 14 days, at pH 5.8 and 7.4, respectively. The release rate increased with decreasing pH, indicating that ATRA-g-PBAE copolymer is a pH-sensitive drug-polymer conjugate.

The release curves show that the release of ATRA-g-PBAE NPs is acid-responsive. ATRA was covalently grafted to the ATRA-g-PBAE copolymer, and its release was controlled by dissociation of ester bonds. At low pH values, ester bonds between drug and polymer are hydrolyzed faster, and grafted ATRA is released at a higher rate. At pH 7.4, release of ATRA is about 42.14% over a period of 21 days, indicating that ATRA-g-PBAE NPs remain stable in physiological condition. When pH is changed to 5.0, amount of released ATRA is up to 66.09%, and ATRA is released more rapidly from the NPs. These results are consistent with the fact that ester linkage degrades faster in acidic solution. These intracellular degradation results demonstrate that ester bonds connecting ATRA are facilely cleaved through hydrolysis within the cancer cells, and free ATRA could be thereby released from NPs. This pH-sensitive system can potentially release conjugated drug at a tumor site either extracellular at acidic pH of tumoral tissues or intracellular, at the acidic pH of the endosomes and the lysosomes.^32^ Therefore, minimum drug loss happens in circulation and extracellular environments while the release in acidic environment of tumor increases, which can enhance the overall therapeutic efficacy.^33^ These results are corroborated by the results obtained in other studies, which indicated effective role of pH-sensitive formulations of NPs in cancer therapy.^22,34^

### Drug release kinetics

The kinetic release model for ATRA-g-PBAE NPs was performed, and coefficient of determination (R_2_) for each model was determined based on the data obtained during *in vitro* release study. Based on our findings, the release data (ATRA release from NPs for 21 days) were kinetically best fitted with the Higuchi square root model at pH 5.8 and 7.4. The slope of the obtained equations in the Higuchi square root model was higher than 0.99 and 0.98, at pH 5.8 and 7.4, respectively. The coefficient of determination (R_2_) for each model at pH 5.8 and 7.4 are summarized in Table 3.

**Table 3:** Squared correlation coefﬁcient values for the ATRA-g-PBAE NPs.

**Mathematical models**	**Squared correlation coefﬁcient (R**_2_ **)**		
	**pH: 5.8**	**pH: 7.4**
First order	0.98	0.91
Hixsone Crowell cube root	0.97	0.90
Zero order	0.93	0.87
Higuchi square root	0.99	0.98
Peppas- Korsmeyer	0.94	0.95

### In vitro cytotoxicity study

**Figure 5:**
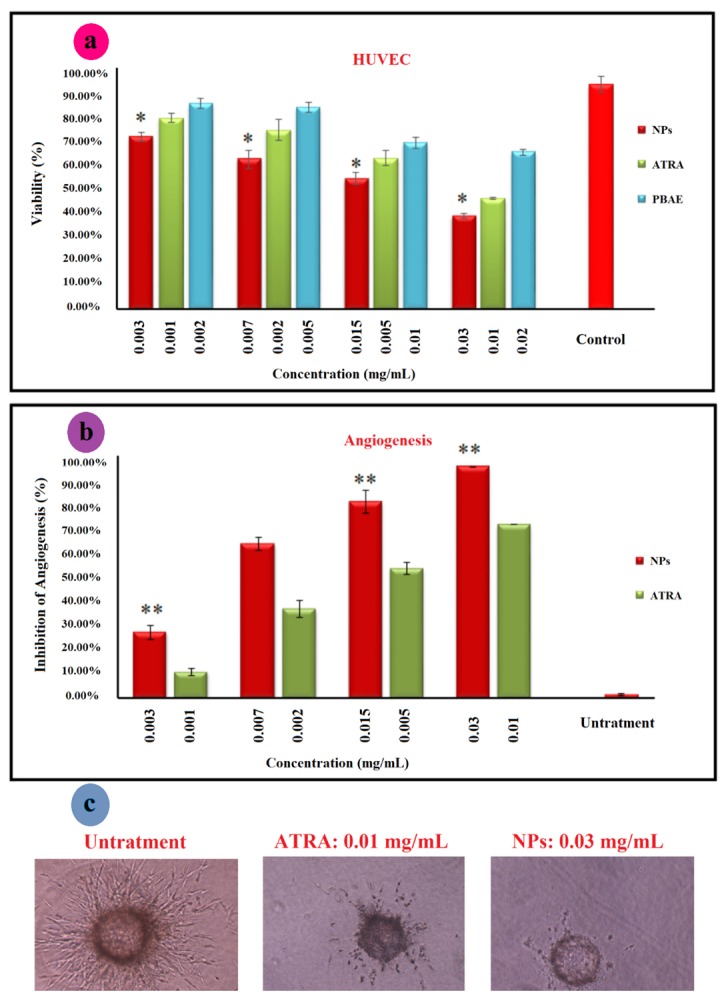
(a) MTT assay for cytotoxicity of ATRA, PBAE and ATRA-g-PBAE NPs on HUVEC cells line; the relative cell viability read for the samples the cell culture medium as a negative control and paclitaxel as positive control (data was not shown) after 48 h of incubation was taken as the reference (100%). (b) anti-angiogenic effect of ATRA and NPs using the 3D collagen-cytodex model. ATR was used as a positive control, and the cell culture media was used as a negative control. Signiﬁcance was calculated by ANOVA (**P ≤ 0.01); Bright field pictures of Cytodex beads for negative control, ATRA and ATRA-g-PBAE NPs (c).

Cytotoxicity has been applied to determine the cytotoxic dose of the compound before anti-angiogenesis studies. Cytotoxicity of ATRA, PBAE, and ATRA-g-PBAE NPs was determined using the MTT cell proliferation assay. The NPs were prepared in four concentrations, and the cytotoxicity of these concentrations was compared with that of pure drug and PBAE, separately. The pure drug and PBAE were used in equal amounts as those presented in the NPs with different concentrations. The results showed that PBAE was not cytotoxic while the drug and NPs were cytotoxic toward HUVEC, (Figure 5a). ATRA and NPs showed a dose-dependent manner and had a considerable higher cytotoxicity effect on HUVEC cells. The IC_50_ (the concentration having 50% cell proliferation inhibition) value of NPs was <0.03 mg/mL (equal to 0.01 mg/mL of ATRA), similar to that of free ATRA.

### Effects on sprout formation of HUVEC in collagen gel (in vitro model)

To determine anti-angiogenic activity of ATRA, PBAE, and ATRA-g-PBAE NPs, we used the 3D collagen-cytodex as an *in vitro* model. In this model, control wells which had not received ATRA, PBAE and ATRA-g-PBAE NPs exhibited a branching pattern of tubular structures 72 h after model design. Pure ATRA and PBAE were used in equal amounts as those in the NPs with different concentrations. The results showed that PBAE in any concentrations and ATRA in concentrations of 0.002 and 0.001 mg/mL could not reach anti-angiogenesis IC_50_ while at dose of 0.005 mg/mL and higher concentrations, ATRA had anti-angiogenic effects. Inhibition of angiogenesis by ATRA at concentrations of 0.01 and 0.005 mg/mL were 75% and 58%, respectively (Figure 5b). NPs indicated the anti-angiogenic activity in concentrations of 0.03, 0.015 and 0.007 mg/mL (equal to 0.01, 0.005 and 0.002 mg/mL of ATRA, respectively) with 100, 85 and 68% inhibition respectively, indicating that the NPs were almost twice more potent than ATRA in inhibition of angiogenesis *in vitro*. While anti-angiogenesis IC_50_ of free ATRA was close to its cytotoxicity IC_50_ and it can be affected by its cytotoxicity effect, anti-angiogenesis IC_50_ of ATRA-g-PBAE NPs was significantly lower than their cytotoxicity IC_50_. Higher potency of NPs can be attributed to improving water solubility and enhancing cellular uptake, which can increase cytoplasmic concentration of ATRA. ATRA can show that both angiogenesis and anti-angiogenesis effects depend on its concentration. Previous studies indicated that in a concentration higher than 1 μM/L, ATRA had an anti-angiogenic effect^35^ whereas in concentrations lower than this could not inhibit angiogenesis and could even stimulate angiogenesis.^36^ Our results for ATRA are in agreement with the previous studies; ATRA amount loaded in NPs was 6.7 to 33.3 μM/L, which could inhibit angiogenesis.

## Conclusion

Developing chemotherapy with nanoplatforms offers a promising strategy for effective cancer treatment. Recently, ATRA, as a potential antitumor drug, has drawn great attention in improving its antitumor activity. In this study, a novel drug-polymer graft from PBAE and ATRA was synthesized, and ATRA-g-PBAE NPs were prepared by the displacement method. RSM has been used to evaluate the effects of the ATRA-g-PBAE copolymer concentration, the surfactant concentration, and the volume ratio of the organic to aqueous phases. Results indicated that all these parameters were effective although the polymer concentration had the highest effect, and only the interaction between polymer and surfactant concentrations had a significant influence on particle size. TEM images showed that NPs had a spherical shape and were monodisperse. The release study showed that drug payload could sustain the release, and ATRA had faster release at endosomal and tumoral pH. While NPs had a similar anti-proliferation effect as ATRA, the anti-angiogenesis study indicated that the NPs were almost twice more potent than ATRA in inhibiting angiogenesis *in vitro* and their anti-angiogenic dose was significantly lower than their cytotoxicity IC_50_ value. In conclusion, ATRA-g-PBAE NPs can be a potential anticancer drug carrier for co-delivery of anticancer drugs with inherent anti-angiogenic effects.

## Ethical Issues

Not applicable.

## Conflict of Interest

Authors declare no conflict of interest in this study.

## Acknowledgments

The authors gratefully acknowledge the Research Council of Kermanshah University of Medical Sciences (Grant Number: 95181) for financial support.
